# Colonization of lettuce rhizosphere and roots by tagged *Streptomyces*

**DOI:** 10.3389/fmicb.2015.00025

**Published:** 2015-02-06

**Authors:** Maria Bonaldi, Xiaoyulong Chen, Andrea Kunova, Cristina Pizzatti, Marco Saracchi, Paolo Cortesi

**Affiliations:** Department of Food, Environmental and Nutritional Sciences, University of MilanMilan, Italy

**Keywords:** biocontrol, *Lactuca sativa*, *Sclerotinia sclerotiorum*, streptomycetes, rhizosphere competence

## Abstract

Beneficial microorganisms are increasingly used in agriculture, but their efficacy often fails due to limited knowledge of their interactions with plants and other microorganisms present in rhizosphere. We studied spatio-temporal colonization dynamics of lettuce roots and rhizosphere by genetically modified *Streptomyces* spp. Five *Streptomyces* strains, strongly inhibiting *in vitro* the major soil-borne pathogen of horticultural crops, *Sclerotinia sclerotiorum*, were transformed with pIJ8641 plasmid harboring an enhanced green fluorescent protein marker and resistance to apramycin. The fitness of transformants was compared to the wild-type strains and all of them grew and sporulated at similar rates and retained the production of enzymes and selected secondary metabolites as well as *in vitro* inhibition of *S. sclerotiorum*. The tagged ZEA17I strain was selected to study the dynamics of lettuce roots and rhizosphere colonization in non-sterile growth substrate. The transformed strain was able to colonize soil, developing roots, and rhizosphere. When the strain was inoculated directly on the growth substrate, significantly more t-ZEA17I was re-isolated both from the rhizosphere and the roots when compared to the amount obtained after seed coating. The re-isolation from the rhizosphere and the inner tissues of surface-sterilized lettuce roots demonstrated that t-ZEA17I is both rhizospheric and endophytic.

## INTRODUCTION

Roots anchor plants in soil, provide uptake of water and nutrients, and mediate numerous interactions with soil organisms. The interface between roots and soil – where most of these interactions take place – is called rhizosphere. This narrow and specific zone is distinct from bulk soil in terms of nutrient availability, pH and presence of a wide variety of microorganisms and invertebrates attracted and influenced by root exudates and rhizodeposits (Hinsinger et al., 2009; Compant et al., 2010; Philippot et al., 2013). Many microbes present in rhizosphere have neutral effect on plants, while others positively or negatively affect host development and health via complex interactions, which we are only beginning to understand (Raaijmakers et al., 2009; Compant et al., 2010; Glick, 2012). Some microorganisms are deleterious as they compete with plants for nutrients or cause disease (soil borne plant pathogens), while others support their hosts by mobilizing nutrients, stimulating growth, and increasing yield or reducing biotic and abiotic stresses, such as mycorrhizal fungi and plant growth promoting bacteria (PGPB; Compant et al., 2010; Aeron et al., 2011; Smith and Smith, 2011).

Plant growth promoting bacteria are gaining more and more attention in modern agriculture, where sustainable and environmentally friendly strategies of crop cultivation increasingly rely on their use as biofertilizers, phytostimulants, or biopesticides. They employ several mechanisms to improve the plant growth, such as synthesis of phytohormones, nitrogen fixation and increasing availability of nutrients by production of siderophores and solubilization of phosphates (Lugtenberg et al., 2002; Compant et al., 2010). Furthermore, special attention is dedicated to biological control agents (BCAs), a group of microorganisms producing a wide variety of biologically active molecules potentially able to inhibit plant pathogens. Antagonism is one of the most common modes of action; here the BCA inhibits or kills pathogens via production of diffusible or volatile antimicrobial compounds and cell wall degrading enzymes. Antagonism is widespread in *Bacillus*, *Pseudomonas,* and *Streptomyces* spp., from which a wide range of biologically active secondary metabolites were isolated (Raaijmakers et al., 2002; Compant et al., 2005).

Despite the optimal performance at laboratory-scale screening tests, PGPB often fail to demonstrate their potential or show inconsistent results in greenhouse and field trials. This variable performance may have different causes, such as reduced or delayed expression of bioactive molecules in the presence of competing microorganisms or lower rhizosphere competence, i.e., poor colonization of root tissues and rhizosphere of the host plant (Lugtenberg et al., 2001; Compant et al., 2010; Ghirardi et al., 2012). To overcome these obstacles, it is essential to understand how PGPB interact with the host plant and with other microorganisms present in soil. Several studies have demonstrated better plant protection when bioactive *Pseudomonas* spp. strains with improved rhizosphere-competence were used (Ghirardi et al., 2012). In recent years, several characters essential for rhizosphere colonization were identified in *Pseudomonas* spp. (Latour et al., 2003; Lugtenberg and Kamilova, 2009), however, similar studies are missing for other beneficial genera of bacteria.

One of such genera is *Streptomyces*, filamentous Gram-positive bacteria commonly inhabiting soil and rhizosphere and renowned for the production of a variety of bioactive secondary metabolites (Loria et al., 2006; Hopwood, 2007). They have been largely exploited in pharmaceutical industry since 1940s (Watve et al., 2001; Lucas et al., 2013), whereas only a few have been developed as commercial products for plant application in agriculture (Yuan and Crawford, 1995; Minuto et al., 2006; Berg, 2009). Streptomycetes have been long considered simply as free-living soil inhabitants, but recently the importance of their complex interactions with plants and other organisms is being uncovered (Seipke et al., 2012). Some of them, such as *S. scabies* or *S. turgidiscabies,* are plant pathogens with broad host range, causing important economic losses especially on tap root and tuber crops, such as potatoes, sweet potatoes, carrots, or beet (Loria et al., 2006; Seipke et al., 2012). On the contrary, many others establish beneficial relationships with host plants as endophytes (Sardi et al., 1992; Coombs and Franco, 2003; Cao et al., 2004). Auxin production was described for endophytic and free living *Streptomyces* in rhizosphere (Coombs et al., 2004; Khamna et al., 2009), while *S. lydicus* augmented the nodulation by *Rhizobium* species in pea plants, increasing iron and molybdenum assimilation as well as root growth (Tokala et al., 2002; Seipke et al., 2012).

Several markers have been developed and adopted to study localization and quantification of PGPB in the rhizosphere; among these, antibiotic resistance has been widely used (Prosser, 1994; Gamalero et al., 2003). Because many of soil microorganisms produce a variety of different antibiotics, it is necessary to determine the specificity of the antibiotic marker selected for the identification of PGPB before its use. Currently, fluorescent markers are gaining increasing popularity for colonization studies (Lu et al., 2004; Cao et al., 2011; Krzyzanowska et al., 2012). Various derivatives of green fluorescent protein (GFP) have been engineered to increase the fluorescence and to overcome the variable expression of the original marker in different species (Errampalli et al., 1999; Gamalero et al., 2003). Enhanced GFP (EGFP) contains numerous silent nucleotide changes in comparison to GFP to maximize its expression in mammalian cells (Haas et al., 1996), and was adopted for use in *Streptomyces* spp., which have a similar codon usage (Sun et al., 1999).

Green fluorescent protein has been utilized to study PGPB colonization of roots and rhizosphere in sterile conditions (Coombs and Franco, 2003; Weyens et al., 2012). These studies provide a basic understanding of the interactions between PGPB and the host plant, but they do not consider the complex interactions *in vivo*. In non-sterile conditions with high microbial diversity, PGPB have to compete with other microorganisms present in the rhizosphere, and in some cases the competition reduced the colonization ability of PGPB (Cao et al., 2011; Hohmann et al., 2012; Weyens et al., 2012). Moreover, the activity and the fitness of the transformed strain need to be controlled following the transformation, as it has been observed that the presence of the transgene may interfere with the biological activity of the studied organism (Nigro et al., 1999; Lübeck et al., 2002; Weyens et al., 2012).

The objective of this work was to get insight into the localization and colonization of a genetically modified *Streptomyces* strain, selected as potential BCA, in lettuce roots and rhizosphere. First, we compared the fitness of the transformed and the corresponding wild-type strains, then we studied the colonization dynamics of the most promising transformed strain in rhizosphere and roots of lettuce plants in non-sterile growth substrate. Finally, we compared the effect of two inoculation methods on the ability of the *Streptomyces* strain to differentially colonize rhizosphere and roots.

## MATERIALS AND METHODS

### TRANSFORMATION OF *Streptomyces* spp.

Five *Streptomyces* strains, potential BCAs against *Sclerotinia sclerotiorum*, were used in this study: CX14W, CX16W, FT05W, SW06W, and ZEA17I. They were maintained at the Plant Pathology Laboratory, Department of Food, Environmental and Nutritional Sciences (DeFENS), University of Milan, and selected previously from a wide collection of actinomycetes isolated from roots of different plants (Sardi et al., 1992; Petrolini et al., 1996; Bonaldi et al., 2011, 2014). *Escherichia coli* strain ET12567 (harboring the helper plasmid pUZ8002), was provided by prof. Flavia Marinelli, University of Insubria, Italy, and was used as donor strain for conjugation. Plasmid pIJ8641, obtained from prof. Mervyn Bibb, John Innes Centre, UK, was maintained in *E. coli* strain DH5α. It carries the EGFP gene under the constitutive *ermE* promoter, an apramycin resistance marker [*aac*(*3*)*IV*], an *oriT/*RK2 region, and a lambda phage chromosomal integration sequence (IntC31; Sun et al., 1999). The strain *S. coelicolor* A3(2) was obtained from F. Marinelli, and used as a reference strain to evaluate transformation efficiency.

Plasmid pIJ8641 was transformed into the donor strain *E. coli* ET12567 (pUZ8002) by rubidium chloride method (Hanahan, 1983) and conjugated into recipient *Streptomyces* strains as previously described (Kieser et al., 2000). Prior to conjugation, the concentration of the *E. coli* donor strain ET12567 containing plasmid pIJ8641 was adjusted to 1 × 10^7^ CFU/mL. The ex-conjugants were selected on the basis of apramycin resistance. The conjugation efficiency was calculated as number of ex-conjugant colonies per number of recipient spores.

Genomic DNA of wild-type and transformed (t-) *Streptomyces* strains was extracted by the CTAB method (Kieser et al., 2000). The amplification of 16S rDNA fragment (expected size 1500 bp) was used to evaluate the quality of DNA in all samples, using PCR primers fD1 (5′-AGAGTTTGATCCTGGCTCAG-3′) and fD2 (5′-ACGGCTACCTTGTTACGACTT-3′; Weisburg et al., 1991), and the following thermal cycling conditions: initial denaturation at 94°C for 1 min, 30 cycles of denaturation at 92°C for 45 s, annealing at 56°C for 30 s and extension at 72°C for 2 min, a final extension at 72°C for 5 min. PCR primers rEGFP-N (5′-CTGGTCGAGCTGGACGGCGACG-3′) and rEGFP-C (5′-CACGAACTCCAGCAGGACCA TG-3′) were designed to amplify the EGFP gene fragment (expected fragment size 700 bp), using the following thermal cycling conditions: initial denaturation at 94°C for 1 min, 30 cycles of denaturation at 92°C for 45 s, annealing at 60°C for 45 s and extension at 72°C for 2 min, a final extension at 72°C for 2 min. The DNA amplification was carried out using PCR thermal cycler (BioRad, USA), performed in a total volume of 25 μL containing 30–50 ng DNA, 0.25 μM each primer, 1 U/μL Go *Taq* DNA polymerase (Promega, USA), 5 μL of 5x Go Taq buffer (Promega, USA), and 0.2 mM of each dNTP. The PCR products were visualized under Gel Doc transilluminator (BioRad, USA) following electrophoresis in 1%(w/v) agarose gel.

For microscopic observations, the transformed *Streptomyces* strains were inoculated on Czapek yeast extract agar (CZY; 35 g/L Czapek-Dox Broth Difco, 2 g/L Yeast Extract Difco, 15 g/L agar). A microscopic cover slide was partially inserted in the medium at the edge of the inoculated strain under the 45° angle to allow the strain to grow on the cover slide. The plates were incubated at 24°C for 5 days. Subsequently, cover slides were removed from the medium and observed by brightfield and epifluorescence microcopy using Olympus BX51 with the FITC filter set (467–498 nm excitation and 513–556 nm emission) to confirm the expression of EGFP in transformants.

### INHIBITION OF *Sclerotinia sclerotiorum* GROWTH *IN VITRO*

The antagonistic activity of wild-type and transformed (t-) *Streptomyces* strains against *S. sclerotiorum* was determined by dual culture assay on CZY agar as described (Bonaldi et al., 2014). Briefly, 10 μL of *Streptomyces* spore suspension (1 × 10^7^ CFU/mL) were inoculated on a 40 mm line two days prior to *S. sclerotiorum* inoculation. An agar-mycelium plug (5 mm diameter), obtained from the edge of an actively growing colony of *S. sclerotiorum* grown on Malt Extract Agar (MEA; 20 g/L Malt Extract, Difco, and 15 g/L agar), was placed at 25 mm distance from the growing *Streptomyces* colony and the plates were incubated for 72 h at 24°C. Plates inoculated with *S. sclerotiorum* only were used as a control. The antagonistic activity was determined by calculating the percentage of growth inhibition of *S. sclerotiorum* compared to the control. The experiment was repeated twice in three replicates.

### MYCELIUM GROWTH AND SPORULATION

The mycelium growth curve of transformed and wild-type strains was determined daily as follows: 20 μL of *Streptomyces* spore suspension (1 × 10^7^ CFU/mL) were transferred into a 50 mL tube containing 20 mL of CZY broth, and incubated at 30°C with 200 rpm constant shaking for 8 days. Each day, 2 mL of liquid culture were removed and spun at 10600 *g* for 10 min and the dry weight of the pellet was repeated twice in three replicates and expressed in g/L.

The sporulation of the strains was measured by plating 1 mL of spore suspension (1 × 10^7^ CFU/mL) on a CZY agar plate and determining the number of spores produced after 6 days of incubation at 30°C (Grantcharova et al., 2005). Five mL of sterile water were added to the Petri plate and the surface of colonies was gently scraped to release the newly formed spores (Kieser et al., 2000). The spore suspension was filtrated through two layers of sterile gauze and the spore concentration (CFU/mL) was quantified by plating serial dilutions of the spore suspension and counting the number of colonies grown after 4 days of incubation at 30°C. The experiment was repeated twice in three replicates.

### PRODUCTION OF SECONDARY METABOLITES

#### Siderophore production

Ten milliliter of Fe-free Czapek solution (300 g/L NaNO_3_, 50 g/L KCl, 50 g/L MgSO_4_ ⋅ 7H_2_O) were mixed with 15 g/L agar, 30 g/L sucrose, 1 g/L K_2_HPO_4_, and 5 g/L yeast extract to prepare the Fe-free Czapek agar medium. Ten microliter of *Streptomyces* spore suspension (1 × 10^7^ CFU/mL) were inoculated in the center of a Fe-free Czapek agar plate and incubated at 30°C for two weeks. Subsequently, the *Streptomyces* colony was overlaid by 15 mL of the Chrome azurol S (CAS) agar (Schwyn and Neilands, 1987; Perez-Miranda et al., 2007). The siderophore production was determined as color change in the overlay medium (from blue to orange) after 24 h of incubation at room temperature. The experiment was repeated twice in three replicates.

#### Chitinase production

The colloidal chitin and the colloidal chitin agar were prepared as described previously (Saima et al., 2013). Ten microliter of *Streptomyces* spore suspension (1 × 10^7^ CFU/mL) were inoculated in the center of colloidal chitin agar plate (chitin as single carbon sources) as a 40 mm line and incubated at 30°C for 10 days. The production of chitinase was determined based on the presence of a clear hydrolysis zone on the agar plate below the colony. The experiment was repeated twice in three replicates.

#### Phosphate solubilization

The phosphate solubilization activity of the *Streptomyces* strains was assessed using a plate assay with National Botanical Research Institute’s Phosphate (NBRIP) medium (Nautiyal, 1999), in which Ca_3_(PO_4_)_2_ is the only phosphate source. Ten microliter of *Streptomyces* spore suspension (1 × 10^7^ CFU/mL) were inoculated in the center of a NBRIP-medium Petri plate and incubated at 30°C for 2 weeks. The phosphate solubilization was determined based on the presence of a clear hydrolysis zone on the agar plate below the colony. The test was repeated twice in three replicates.

#### Indole-3-acetic acid (IAA) synthesis

The IAA production was determined as described previously (Bric et al., 1991; Bano and Musarrat, 2003). In brief, 10 μL of *Streptomyces* spore suspension (1 × 10^7^ CFU/ml) were incubated with constant shaking at 5 *g* in 5 mL CZY broth added with 500 μg/mL tryptophan (Sigma, USA) in the dark at 30°C for 10 days. Two mL of the liquid culture were centrifuged for 10 min at 18000 *g*. One mL of the supernatant was mixed with 50 μL 10 mM orthophosphoric acid and 2 mL of Salkowski reagent (1 mL of 0.5M FeCl_3_ in 50 mL of 35% HClO_4_). The tubes were incubated at room temperature for 30 min. The development of pink color indicated the IAA production, which was quantified by spectrophotometer at 530 nm. The experiment was repeated twice in three replicates.

### SOIL, ROOT, AND RHIZOSPHERE COLONIZATION BY t-ZEA17I

The transformed ZEA17I strain (t-ZEA17I) was grown on CZY medium containing 50 mg/L of apramycin at 24°C for 10 days. Spores were collected in 10% sterile glycerol and filtered through two layers of gauze. The concentration was determined and the spore suspension was stored at -20°C.

#### Bulk soil colonization

Prior to colonization studies, the presence of naturally occurring apramycin-resistant streptomycetes in non-sterilized Irish and Baltic peat-based growth substrate (Vigorplant, Italy) was assessed. A portion of the substrate was resuspended in sterile water and plated on water agar medium (WA) containing 15 g/L agar, 25 mg/L nalidixic adic, 50 mg/L apramycin, 50 mg/L nystatin, and 50 mg/L cycloheximide. Plates were incubated for 7 days at 24°C and the presence of apramycin-resistant streptomycete colonies was visually checked.

The growth substrate was placed in a polystyrene seed tray (48 cm^3^/cell) and watered with tap water. In every cell, t-ZEA17I was uniformly distributed on the top of the substrate adding 1 mL spore suspension (1 × 10^7^ CFU/mL). The growth substrate was incubated in a growth chamber (24°C, 55% relative humidity and 15 h photoperiod) and watered every 2–3 days with tap water. t-ZEA17I was re-isolated 4 h (day 0), 10, 20, and 30 days after inoculation (dai) in six replicates. The entire amount of growth substrate in the cell was collected and weighed. The substrate was mixed to homogenize the inoculum and divided in two identical parts. One part was incubated in the oven at 50°C and the dry weight was determined. The other part was stirred in sterilized water (1:10 substrate fresh w/v) for one hour and serial dilutions were plated on WA. Plates were incubated for 7 days at 24°C and streptomycete colonies were counted. The t-ZEA17I concentration was expressed as CFU/g of growth substrate dry weight.

#### Plant inoculation

Ice queen lettuce seedlings (*Lactuca sativa* var. *capitata*, Iceberg group, Semeurop, Italy) were grown in polystyrene seed trays, as described previously. Seeds were surface sterilized in 0.7% sodium hypochlorite for 5 min and rinsed three times in sterile water. Two methods were used to inoculate the t-ZEA17I strain. In the growth substrate inoculation method, 1 mL spore suspension (1 × 10^7^ CFU/mL) was uniformly distributed in every cell on the top of the growth substrate. In the seed coating method, 50 seeds were soaked in 1 mL of t-ZEA17I spore suspension (1 × 10^7^ CFU/mL) and left to dry under the laminar flow hood. One seed for each cell of the tray was sown and the seedlings were incubated and watered as described previously.

To determine the inoculum load t-ZEA17I was re-isolated from seeds and growth substrate after inoculum application. In case of the growth substrate inoculation method, the t-ZEA17I strain was re-isolated four hours after soil inoculation as described above for bulk soil, and its amount was expresses as CFU/g of growth substrate dry weight. For the seed coating method, 10 randomly collected seeds were incubated for 30 min in 1 mL of sterile 0.9% NaCl and serial dilutions were plated on WA medium in six replicates. Following incubation at 24°C for 7 days the t-ZEA17I colonies were counted and the amount was expressed first as CFU/seed and then recalculated as CFU/g of growth substrate dry weight.

#### t-ZEA17I re-isolation from rhizosphere and root tissues

The t-ZEA17I strain was re-isolated 10, 20, and 30 days after sowing from rhizosphere and root tissues of six lettuce seedlings, equal to number of replicates. Seedlings with root system were carefully taken off the cell and the bulk soil was removed by gently shaking the plants (Bulgarelli et al., 2012).

For the rhizosphere analysis, each seedling was cut at base and the roots were vortexed two times for 15 s in 1–3 mL (volume varying according to period of sampling) of sterilized 0.9% NaCl and 0.02% Silwet L-77 (washing solution). The roots were removed and the suspension was filtered through a 300 μm nylon mesh to obtain the rhizosphere soil and its dry weight was determined. The suspension was centrifuged at 10600 *g* for 10 min and the pellet was resuspended in 0.5–1.5 mL of washing solution and plated in serial dilutions on WA medium. The plates were incubated at 24°C for 7 days. The t-ZEA17I colonies were counted and the concentration was expressed as CFU/g of rhizosphere dry weight.

For inner root tissues analysis, the roots were surface sterilized with propylene oxide for one hour (Sardi et al., 1992). Then, they were washed in washing solution and 1/10 of the total volume was plated on WA medium to verify the absence of contaminants. Subsequently the roots were finely homogenized in 1–3 mL washing solution, let to macerate for one hour and the suspension was plated in serial dilutions. The t-ZEA17I concentration was determined as described before and expressed as CFU/g of roots dry weight.

### STATISTICAL ANALYSES

All analyses were done using R software, version R3.0.2. (R Core Team, 2013). The statistical differences between data of transformed and wild-type strains in *S. sclerotiorum* growth inhibition, sporulation, and IAA production were compared by a Student’s *t*-test (*P* = 0.05). The percent data were arcsine root-squared transformed. The soil, root, and rhizosphere colonization data were submitted to ANOVA, followed by a Tukey *post hoc* test for multiple comparison (*P* = 0.05), using the TukeyC package (Faria et al., 2013).

## RESULTS

### TRANSFORMATION OF *Streptomyces* spp. WITH PLASMID pIJ8641

**FIGURE 1:**
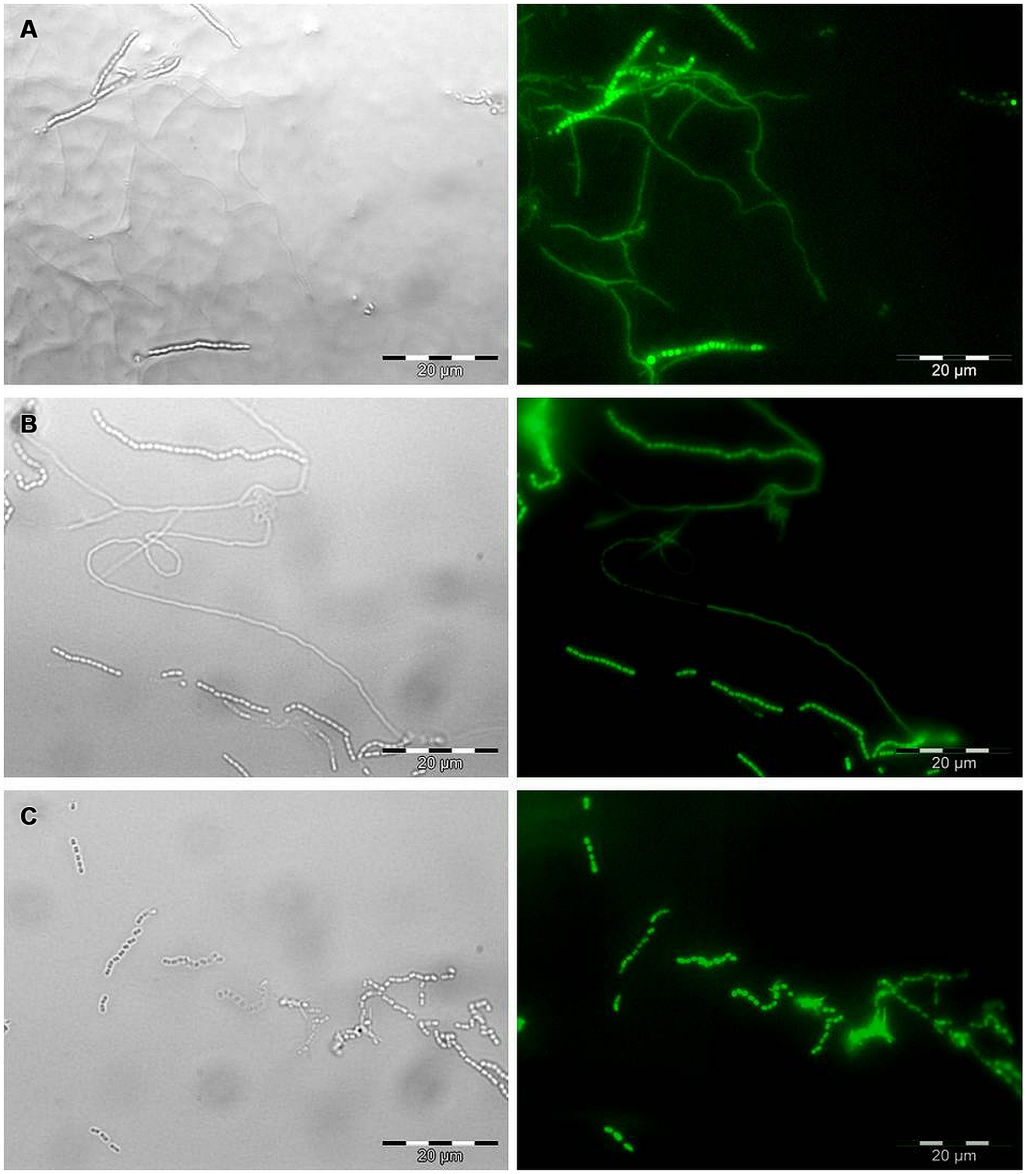
**Microscopic observation of EGFP expression in transformed *Streptomyces* strains by bright field (left) and epifluorescence microscopy (right).** Mycelium and spore chains of **(A)** the strain t-ZEA17I, **(B)** the strain t-FT05W, and **(C)** spore chains of the strain t-CX16W. Scale bar, 20 μm.

All six strains, including *S. coelicolor* A3(2) were transformed with the pIJ8641 plasmid harboring the EGFP gene under a constitutive promoter and apramycin resistance. Conjugation efficiency varied among strains: four strains, CX14W, SW06W, CX16W, and FT05W showed conjugation efficiency similar to *S. coelicolor* A3(2), while one strain, ZEA17I conjugated with lower efficiency (**Table 1**). The EGFP gene was detected in transformed strains (data not shown), and its expression was confirmed by fluorescence microscopy observing a strong green fluorescence in all transformants following exposition to fluorescent light (**Figure 1**), while the corresponding wild-type strains did not fluoresce.

**Table 1:** Conjugation efficiencies of five *Streptomyces* spp. strains and *S. coelicolor* A3(2) with the pIJ8641 plasmid.

Strain	Recipient strain (CFU/mL)	Conjugation efficiency (CFU/recipient strain)
*S. coelicolor* A3(2)	1 × 10^8^	9.10 × 10^-6^
CX14W	1 × 10^8^	4.64 × 10^-5^
CX16W	1 × 10^10^	6.88 × 10^-6^
FT05W	1 × 10^9^	1.60 × 10^-6^
SW06W	1 × 10^8^	3.13 × 10^-5^
ZEA17I	1 × 10^9^	5.81 × 10^-8^

### EFFECT OF THE TRANSFORMATION ON STRAIN FITNESS

**FIGURE 2:**
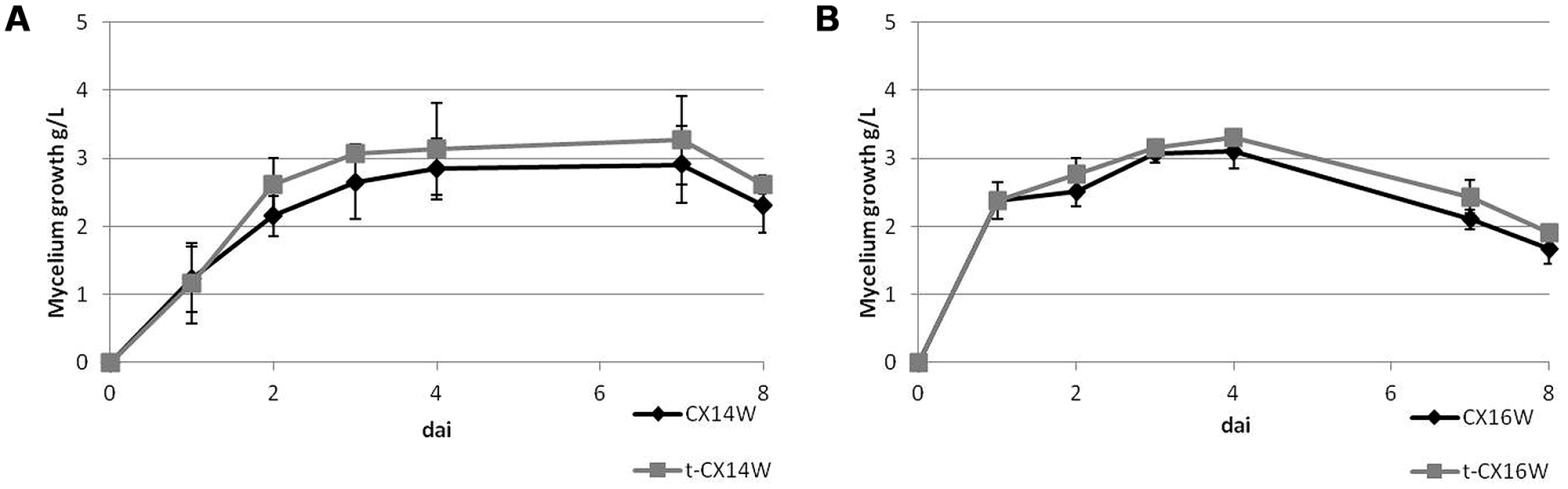
**Mycelium growth of the wild-type and transformed *Streptomyces* spp. strains, **(A)** CX14W and **(B)** CX16W.** Vertical bars represent SE (*N* = 3).

Following the transformation, the fitness of transformants was evaluated in terms of mycelium growth and sporulation, inhibition of *S. sclerotiorum* mycelium growth, and production of selected secondary metabolites. All the transformed strains showed similar mycelium growth and sporulation to their corresponding wild-type strains (**Figure 2** – for simplicity, the growth curves for only two strains were reported, **Table 2**). The transformants inhibited *S. sclerotiorum* radial growth from 66 to 81%, and no significant differences were observed between wild-type and transformed strains. Finally, no significant differences were detected in siderophore, auxin, chitinase production, and phosphate solubilization (**Tables 2** and **3**).

**Table 2:** Sporulation, auxin production, and inhibition of *Sclerotinia sclerotiorum* mycelium growth of the wild-type and transformed (t-) *Streptomyces* spp.

Strain	Sporulation (10^9^ CFU/mL)		Auxin production (μg/mL)		*S. sclerotiorum* inhibition (%)	
CX14W	7.47 ± 3.22^1^	*P^2^ *=* 0.229	5.82 ± 0.44	*P = 0.278	78.80 ± 1.70	*P = 0.072
t-CX14W	6.80 ± 4.91		5.11 ± 0.35		73.00 ± 2.28	
CX16W	4.23 ± 6.58	*P = 0.178	5.87 ± 0.86	*P = 0.872	68.99 ± 0.98	*P = 0.091
t-CX16W	5.55 ± 1.61		6.03 ± 0.24		65.87 ± 1.33	
FT05W	1.45 ± 3.69	*P = 0.242	6.04 ± 0.60	*P = 0.207	67.59 ± 0.68	*P = 0.684
t-FT05W	1.37 ± 4.56		7.11 ± 0.21		67.13 ± 0.85	
SW06W	2.27 ± 1.15	*P = 0.205	7.32 ± 0.66	*P = 0.588	80.21 ± 1.34	*P = 1.000
t-SW06W	0.78 ± 1.67		6.88 ± 0.28		80.21 ± 1.34	
ZEA17I	0.47 ± 0.23	*P = 0.417	7.56 ± 0.59	* *P =* 0.690	74.80 ± 1.76	*P = 0.069
t-ZEA17I	1.63 ± 1.15		6.84 ± 0.38		80.75 ± 1.51	

*^1^Mean value followed by SE. ^2^*P*-values of the Student’s *t*-test pairwise comparisons.*

**Table 3:** Phosphate solubilization, chitinase, and siderophore production of the wild-type and transformed (t-) *Streptomyces* spp.

Strain	Phosphate solubilization	Chitinase production	Siderophore production


CX14W	+^1^	+	+
t-CX14W	+	+	+
CX16W	+	+	+
t-CX16W	+	+	+
FT05W	+	+	-
t-FT05W	+	+	-
SW06W	+	+	-
t-SW06W	+	+	-
ZEA17I	+	+	-
t-ZEA17I	+	+	-

*^1^+indicates activity. – indicates no activity.*

### COLONIZATION DYNAMICS OF THE TRANSFORMED ZEA17I IN BULK SOIL

In this study, apramycin was used as a marker to identify the transformed t-ZEA17I strain. However, to be able to use it in greenhouse experiments, we first checked for the presence of naturally occurring apramycin-resistant *Streptomyces* spp. in the growth substrate, but none were detected. Therefore non-sterilized growth substrate was used in following experiments.

The colonization dynamics of t-ZEA17I in bulk soil showed that the initial inoculum, 1.16 × 10^8^ CFU/g dry weight added to the soil, was recovered at 3.85 × 10^7^ CFU/g dry weight four hours after inoculation (0 dai). The t-ZEA17I amount decreased significantly within the first 10 days and thereafter it remained stable up to 30 days at 2.18 × 10^4^ CFU/g dry weight (**Table 4**).

**Table 4:** Colonization dynamics in bulk soil by transformed *Streptomyces* ZEA17I.

Inoculation method	Bulk soil (CFU/g dry weight)			
	0 dai^1^	10 dai	20 dai	30 dai
Growth substrate	3.85 × 10^7^a^2^	1.25 × 10^4^b	1.54 × 10^4^b	2.18 × 10^4^b

*^1^dai = days after inoculation. ^2^Tukey *post hoc* test; means in a row with the same letters are not significantly different (*P* = 0.05).*

### COLONIZATION OF LETTUCE RHIZOSPHERE AND INNER ROOT TISSUES BY THE TRANSFORMED ZEA17I

The *Streptomyces* strain t-ZEA17I was inoculated with two different methods: as a spore suspension distributed on soil surface and as seed coating. The colonization dynamics of rhizosphere and inner root tissues of lettuce seedlings differed between the two methods.

In the rhizosphere, the concentration of the t-ZEA17I strain remained similar to the inoculated amount during the first 20 dai with either method. When t-ZEA17I was distributed on top of the growth substrate, a significant increase in concentration 30 dai was observed. In the case of the seed coating, after a slight increase within the first 10 days, the final amount was not significantly different from the initial inoculum (**Table 5**).

**Table 5:** Colonization dynamics of *Lactuca sativa* var. *capitata* rhizosphere by transformed *Streptomyces* ZEA17I strain according to two inoculation methods.

Inoculation method	Rhizosphere (CFU/g dry weight)			
	0 dai^1^	10 dai	20 dai	30 dai
Growth substrate	2.51 × 10^6^ b^2^	2.72 × 10^7^ ab	3.07 × 10^7^ a	3.80 × 10^7^ a
Seed coating	1.28 × 10^6^ ab	2.01 × 10^6^ a	9.85 × 10^5^ ab	1.19 × 10^5^ b

*^1^dai = days after inoculation. ^2^ Tukey *post hoc* test; means in a row with the same letters are not significantly different (*P* = 0.05).*

Similarly, we studied the dynamics of t-ZEA17I colonization in the inner tissues of lettuce roots. First, we ruled out the possible external root contamination due to ineffective sterilization, and no *Streptomyces* colonies were detected. The t-ZEA17I strain was re-isolated from inner root tissues of surface-sterilized roots independently of the inoculation method, confirming its ability to endophytically colonize lettuce roots. The concentration of t-ZEA17I declined steadily through time, however, this reduction was not significant with either inoculation method (**Table 6**).

**Table 6:** Colonization dynamics of *L. sativa* var. *capitata* inner root tissues by transformed *Streptomyces* ZEA17I strain according to two inoculation methods.

Inoculation method	Roots (CFU/g dry weight)		
	10 dai^1^	20 dai	30 dai
Growth substrate	1.94 × 10^7^ ns^2^	1.45 × 10^6^ ns	2.36 × 10^5^ ns
Seed coating	3.93 × 10^5^ ns	2.23 × 10^5^ ns	1.39 × 10^4^ ns

*^1^dai = days after inoculation. ^2^ns, ANOVA not significant (*P* > 0.05).*

Finally, we compared the two inoculation methods to get a further insight into whether one of them could improve the survival and colonization rates of t-ZEA17I in lettuce rhizosphere and roots. In the rhizosphere, significantly more t-ZEA17I was re-isolated at all sampling times using the growth substrate inoculation rather than seed coating (*P* = 0.0037, 0.0389, and 0.0005, for sampling time 10, 20, and 30 dai, respectively). Similarly, in roots, significantly higher concentration of t-ZEA17I was re-isolated using the growth substrate inoculation at 20 and 30 dai (*P* = 0.0415 and *P* = 0.0604, respectively). However, in spite of higher strain amounts present in roots using the growth substrate inoculation method, not all seedlings were colonized. Indeed, we failed to re-isolate t-ZEA17I from roots of three seedlings (one seedling at 20 dai and two seedlings at 30 dai), whereas, using the seed coating method, all roots were endophytically colonized.

## DISCUSSION

Plant beneficial bacteria have a great potential in agriculture as PGPB and BCAs and reports about successful control of plant diseases are increasing. However, application of these microbial agents in field often fails to achieve the expected results, which could be due to lack of knowledge about their biology and interactions with the host plant, the pathogens, and other microorganisms in the rhizosphere. Therefore, there are increasing attempts to study these complex interactions that take place in the rhizosphere (Gamalero et al., 2003; Compant et al., 2010).

Our aim was to study spatio-temporal dynamics of colonization of lettuce roots and rhizosphere by *Streptomyces* spp. with biological control potential, to better understand if and how they inhabit the rhizosphere and colonize plant roots. We selected five *Streptomyces* strains on the basis of their strong *in vitro* antagonism against the major soil-borne pathogen of horticultural crops, *S. sclerotiorum* (Bonaldi et al., 2014), and we transformed them with the pIJ8641 plasmid harboring apramycin resistance marker and EGFP gene under a strong constitutive promoter (Sun et al., 1999). The conjugation efficiency varied, and for most strains it was comparable to the reference strain *S. coelicolor* A3(2). The pIJ8641 plasmid integrates at the chromosomal attachment site for the temperate phage φC31, which may result in disruption or alteration of fitness and biological activity of the transformed strains. Indeed, decrease or loss of biological activity was detected after GFP-transformation of various BCAs, e.g., *Pseudomonas putida*, *Metschnikowia pulcherrima,* or *Clonostachys rosea* (Nigro et al., 1999; Lübeck et al., 2002; Weyens et al., 2012). We compared several traits important for biological control and plant growth promotion of transformed and wild-type strains, before studying their interactions with the host plant. None of the transformed strains showed altered growth or sporulation, which could have conferred a disadvantage in plant root and rhizosphere colonization. All transformants retained the ability to suppress growth of *S. sclerotiorum in vitro*, therefore they will also be used for studying their interactions with the pathogen and the mechanisms of biological control *in vivo*. Moreover, we compared the expression of some of the most common traits involved in plant growth promotion and biological control (Brader et al., 2014), such as production of auxins, siderophores and lytic enzymes, and no change in performance between the wild type and the transformants was observed.

We chose the most promising transformed strain, t-ZEA17I, for pilot studies of lettuce roots and rhizosphere colonization. We intentionally used a non-sterile growth substrate to simulate competition with natural microflora and evaluate the competitiveness of the inoculated *Streptomyces* strain exploiting the apramycin resistance for its identification among soil microorganisms. In absence of the host plant, we confirmed that t-ZEA17I freely survives in soil, although we observed a sharp decrease in its density within the first 10 days after bulk soil inoculation. Similar dynamics for introduced microbial population in non-sterile soil are already known, attributed to scarcity of available nutrients and adverse biotic and abiotic factors (van Veen et al., 1997). However, following the initial fall in population density, the t-ZEA17I population remained stable for up to 30 days, probably establishing an equilibrium with the indigenous microflora as described previously (Yuan and Crawford, 1995; Merzaeva and Shirokikh, 2006). In the presence of the lettuce plant, we did not detect in the rhizosphere the initial rapid decrease in t-ZEA17I amount that was observed in bulk soil. On the contrary, its concentration augmented when applied directly on the growth substrate. It is possible that t-ZEA17I was chemoattracted to the rhizosphere of the growing seedling, where it quickly established a stable interaction with the host plant roots. Indeed, the presence of a host plant may greatly affect the survival of PGPB, as was observed, i.e., for the sharp decline in *Azospirillum brasilense* population after removal of inoculated plants (Bashan et al., 1995).

Different strategies are being used for studying BCAs and PGPB in the rhizosphere. Their localization in roots and seeds rely on microscopic tools exploiting fluorescent markers, which give a fundamental insight into the spatial distribution of the microorganism along and inside the growing root, but do not quantify the microbial amounts and their dynamics (Coombs and Franco, 2003; Olivain et al., 2006; Compant et al., 2010). Additionally, studying the dynamics of colonization by beneficial microorganisms exploits the strain identification mostly by natural or introduced antibiotic resistance and its quantification by dilution plating (Gamalero et al., 2003, 2005). Here, we quantified the t-ZEA17I in roots and rhizosphere through the introduced antibiotic resistance for its identification, to understand if it can inhabit soil in competitive concentrations in comparison to the indigenous microflora. t-ZEA17I was detected in the inner root tissues of growing seedlings already 10 days after inoculation at high concentrations. Indeed, Coombs and Franco (2003) demonstrated that the EGFP-tagged endophytic *Streptomyces* sp. strain EN27 rapidly colonizes the wheat embryo, as it was detected in developing roots as early as 24 h after inoculation. Re-isolation of t-ZEA17I from the rhizosphere and the inner tissues of surface-sterilized roots indicates that it is both rhizospheric and endophytic, although it is not known if its localization affects its potential for biocontrol and plant growth promotion. It has been hypothesized that endophytic bacteria form more stable interactions with plants, rather than rhizospheric or epiphytic bacteria (Compant et al., 2010; Malfanova et al., 2011).

Finally, we tested how different methods of inoculation influence the t-ZEA17I colonization ability. When it was distributed directly on the growth substrate, higher concentration was detected in roots, however, we could not re-isolate the strain from all inoculated plants. On the contrary, when the seed coating method was used, less propagules were recovered but all plants were endophytically colonized. It is possible that in case of seed coating, t-ZEA17I is more closely associated with the growing seedling, which increases its probability to internally colonize root tissues. In roots, we observed progressive decline in its concentration using either inoculation method. Although the total amount of t-ZEA17I increased at different sampling times (data not shown), the increase in lettuce root biomass was probably higher than the strain growth, thus resulting in lower strain concentration per g of root. To ensure that t-ZEA17I colonizes roots in effective concentrations, and to prevent its decline to undetectable levels, studies assessing optimal amount of inoculum are needed. Moreover, it is possible that strains colonize only certain root zones (Gamalero et al., 2004, 2005). Therefore, further studies are needed to establish which zones of the plant roots are colonized by t-ZEA17I and ultimately how it interacts with the plant in presence of *S. sclerotiorum,* to evaluate its biological control activity *in vivo*.

## Conflict of Interest Statement

The authors declare that the research was conducted in the absence of any commercial or financial relationships that could be construed as a potential conflict of interest.
